# A New Method of Dissection

**Published:** 1860-10

**Authors:** 


### A NEW METHOD OF DISSECTION.

A great desideratum in anatomy is, to obtain an exact idea of the real position of the internal organs. This, however, is far from being the case in dissections by the common method; since every section made on the body, rendered flabby and unelastic by death, produces a corresponding deformation; the soft part contract, and nothing but an approximative idea can be formed of the relative position of the exposed parts before the operation. in order to obviate this inconvenience, Dr. Pirogoff, of Russia, has had the ingenious idea of subjecting the body, before dissection, to a cold of eight degrees centrigrade, (16 Fahr.,) for the space of three days. By this means the body acquires a hardness like that of wood, its organs retaining, at the same time, their relative sizes, since the moisture they contain increases by congealation, and thus counteracts the contraction which the solids would otherwise undergo.

The body in this state is subjected to the circular saw, which will cut off slices of the thickness of a frank piece with the greatest nicety, either longitudinally, transversely, or along the axis of the member. By this means Dr. Pirogoff has been enabled to publish an anatomical atlas of every part of the human body, seen under three different aspects. In order to copy out a section obtained in the manner described, Dr. Pirogoff passes lightly over the frozen surface with a warm sponge; the surface is thus thawed for an instant, but a transparent film of ice is immediately afterwards formed over it. A pane of glass with lines drawn upon it crossing each other at right angles, so as to form so many squares, is then laid on the icy film, and the surface copied out upon paper also divided into squares like the glass.

By this means the greatest precision is attained. The principle of refrigeration has been carried still further by the ingenious inventor, who by exposing a body to a cold of 18 degrees centigrade, (4 Fahr.,) reduces it to the consistency of stone, and then operates upon it like a sculptor, with a chisel and mallet, laying all the viscera open without in the least degree injuring them. It is thus he has been enabled to ascertain that the cavities of the mouth, nose, the typanum of the ear, and those of the respiratory organs, are the only ones which enclose air, and that everywhere else the surface of all parts of the body adhere immediately to the membranes enveloping the organs they contain; so that however apparently dissimiliar their surface of contact may be, there is still no empty space between them.

---

☞ The friends of the late Dr. Isaacs will be gratified to learn that a bust of this lamented physician is in preparation by Mr. Thomas Cooper, of this city. The mould was taken after death, which gives a somewhat cadaveric appearance to the features, but otherwise they are strikingly accurate.

